# Uncertainty-Aware Depth Network for Visual Inertial Odometry of Mobile Robots

**DOI:** 10.3390/s24206665

**Published:** 2024-10-16

**Authors:** Jimin Song, HyungGi Jo, Yongsik Jin, Sang Jun Lee

**Affiliations:** 1 Division of Electronic Engineering, Jeonbuk National University, 567 Baekje-daero, Deokjin-gu, Jeonju 54896, Republic of Korea; jimin_song@jbnu.ac.kr (J.S.); hygijo@jbnu.ac.kr (H.J.); 2 Daegu-Gyeongbuk Research Center, Electronics and Telecommunications Research Institute (ETRI), Daegu 42994, Republic of Korea; yongsik@etri.re.kr

**Keywords:** simultaneous localization and mapping, visual-inertial odometry, depth estimation, uncertainty estimation, parking lot dataset

## Abstract

Simultaneous localization and mapping, a critical technology for enabling the autonomous driving of vehicles and mobile robots, increasingly incorporates multi-sensor configurations. Inertial measurement units (IMUs), known for their ability to measure acceleration and angular velocity, are widely utilized for motion estimation due to their cost efficiency. However, the inherent noise in IMU measurements necessitates the integration of additional sensors to facilitate spatial understanding for mapping. Visual–inertial odometry (VIO) is a prominent approach that combines cameras with IMUs, offering high spatial resolution while maintaining cost-effectiveness. In this paper, we introduce our uncertainty-aware depth network (UD-Net), which is designed to estimate both depth and uncertainty maps. We propose a novel loss function for the training of UD-Net, and unreliable depth values are filtered out to improve VIO performance based on the uncertainty maps. Experiments were conducted on the KITTI dataset and our custom dataset acquired from various driving scenarios. Experimental results demonstrated that the proposed VIO algorithm based on UD-Net outperforms previous methods with a significant margin.

## 1. Introduction

**Figure 1:**
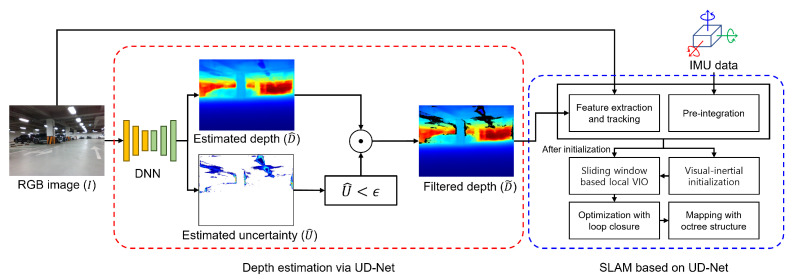
Proposed visual–inertial odometry pipeline based on UD-Net. The pipeline is divided into two main processes: depth estimation for the RGB image by UD-Net, indicated by a red round box, and VIO based on the estimated depth map, indicated by a blue round box. In the depth map, blue indicates closer distances, while red represents farther distances.

Recent advancements in robotics have enabled the utilization of mobile robots across various industries with ongoing developments aimed at further enhancing these technologies and expanding their application fields. For instance, mobile robots are increasingly being applied in extreme environments, such as in rescue missions [1] and space exploration [2]. To execute the challenging tasks required in these fields, such as obstacle avoidance [3] and path planning [4], fast and accurate environmental perception technologies are essential. A representative example of such technology is simultaneous localization and mapping (SLAM), which involves perceiving the surrounding environment to create a map while simultaneously estimating the current location of the robot within the map. For localization, visual markers [5] or radio frequency identification [6] are used in indoor environments, while global positioning system technology [7] is utilized in outdoor settings. However, in certain environments such as those mentioned [1,2], relying on these external elements is not feasible and necessitates the use of the onboard sensors of the robot. Therefore, recent SLAM techniques propose using IMU to measure acceleration and angular velocity for estimating positional changes. These techniques often incorporate additional sensors to compensate for the inherent noise characteristics of IMU. Light detection and ranging (LiDAR) is a remote sensing technology that measures distance with high accuracy by emitting light toward a target and detecting the reflected light with optical sensors. While LiDAR-based SLAM techniques demonstrate high accuracy in indoor environments, they struggle in environments with low reflectivity medium, open roads where point cloud features are less distinct, or under adverse weather conditions such as rain or snow. Therefore, various VIO algorithms, which use cameras and IMUs to estimate the trajectory of mobile robots, have been proposed recently. However, VIO algorithms have continued to demonstrate lower odometry estimation performance compared to LiDAR-based methods, which directly utilize accurate 3D information. As shown in Figure 1, we aim to propose a VIO algorithm that maximizes the advantages of deep learning-based depth estimation to achieve performance comparable to LiDAR-based methods.

Three-dimensional information cannot be directly obtained from a camera sensor, so distance information is often derived from feature matching and triangulation using stereo images or sequences of images. However, distance information obtained through these methods suffers from scale ambiguity and has a trade-off between the effective depth range and estimation accuracy depending on the distance between the two cameras. Micro-electro-mechanical systems-based small LiDAR, which are packaged with cameras as a module, offer high resolution but have the drawbacks of a shorter depth range and relatively lower distance measurement accuracy compared to more expensive mechanical LiDAR systems. Recently, there has been a trend toward applying monocular depth estimation, which leverages deep neural networks (DNNs) to estimate pixel-level distance from a single image, as an alternative to distance measurement sensors in VIO. However, challenges remain in training networks for depth estimation, such as addressing the inherent scale ambiguity, improving network capability efficiently, and acquiring high-quality datasets to enhance generalization performance. In this paper, we propose a network architecture and loss function designed to improve reliability by identifying regions of high uncertainty, where estimation errors are more likely to occur.

Ideally, we would hope that all estimations from deep learning models are error-free, but this is practically impossible. Therefore, estimating uncertainty is necessary to determine the reliability of the estimation results. In deep learning, two primary types of uncertainty are aleatoric uncertainty from noise in the training data and epistemic uncertainty from ambiguity in selecting the most appropriate model structure and parameters for a given task. Such types of uncertainty can also be observed in depth estimation tasks. The ground truth depth maps for training depth networks are generated by projecting 3D point clouds obtained from high-performance LiDAR onto the image plane. For this method, inaccurately projected points due to the viewpoint difference between the LiDAR and camera partially occur, leading to uncertainty caused by this noise of ground truth data. This method provides pixel-level distance with high accuracy in most regions, but it has the disadvantage of lower spatial resolution compared to RGB images from cameras. From the perspective of supervised learning-based methods, uncertainty arises in finding the appropriate model architecture and parameters for depth estimation, as it is not possible to train and evaluate the entire element of predicted results. In this paper, we define a ground truth uncertainty map that enables the direct learning of the uncertainties arising from errors encountered during the depth estimation process. We also apply a filtering technique based on the uncertainty map to enhance the reliability of depth estimation and demonstrate that utilizing the filtered depth map improves the performance of VIO. To evaluate the proposed method, we utilized the KITTI dataset [8], a public road-driving dataset, along with a custom dataset collected from an underground parking lot environment. Across both datasets, our proposed method demonstrated not only improved depth estimation accuracy compared to existing supervised learning approaches but also showed that uncertainty estimation can enhance the overall reliability. Furthermore, by incorporating the reliability-enhanced filtered depth map into the VIO pipeline, we achieved significant improvements in odometry estimation performance. The key contributions of our work are outlined as follows:

- We designed a network named UD-Net, which is a straightforward DNN architecture that uses a shared encoder–decoder structure to estimate both the depth of each pixel in the RGB image and the uncertainty of depth estimation. In contrast to existing research on uncertainty estimation, we propose the uncertainty of depth estimation that allows the network to directly learn regions where errors are likely to occur during the depth estimation process. For training UD-Net, we introduce a depth loss based on the estimated uncertainty and an uncertainty loss based on the estimated depth, which is specifically designed for training UD-Net.
- We integrate UD-Net with the feature-based VIO algorithm [9] to propose a novel VIO algorithm which is robust for the unavoidable errors of the depth network.
- Using the public KITTI dataset, we demonstrated the improved performance of depth estimation achieved by our proposed pipeline. We acquired and processed an underground parking lot dataset to demonstrate that our approach not only improves depth estimation performance but also enhances VIO performance.

## 2. Related Work

Supervised learning-based depth estimation is a method that optimizes the model to reduce the value of the loss function between the estimated depth map and the ground truth depth map. It is considered the most effective pipeline for addressing the inherently ill-posed problem of depth estimation, which involves resolving scale ambiguity from a single image while maintaining high estimation accuracy. In this field, the scale-invariant log error (SIlog) proposed by Eigen et al. [10] is commonly used as a fundamental loss function. Eigen et al. [10] demonstrated that while the global scale of an image is important, incorporating SIlog as a loss function to reflect the relationships between pixels in the estimated depth map enhances depth estimation performance. In this work, we designed a novel loss function for uncertainty-aware depth estimation based on SIlog. Recently, various supervised learning-based algorithms have been proposed, including novel network architectures and approaches such as ordinal regression, which redefine the problem by focusing on relative order. Lee et al. [11] proposed the local planar guidance (LPG) layer based on the geometric assumption that adjacent regions in the image are projected from the same plane. Yuan et al. [12] proposed an algorithm that divides the image into hierarchical windows and estimates potential based on color and depth information between adjacent pixels within each window. Bhat et al. [13] addressed depth estimation as an ordinal regression problem and proposed a method for estimating adaptive bins. We compare the depth estimation performance of our UD-Net with three recently proposed supervised learning algorithms [11,12,13]. Despite continuous improvements in depth estimation performance through various approaches, there has been insufficient focus on enhancing the reliability of depth networks. Therefore, this paper proposes a network capable of simultaneously estimating uncertainty, considering its application in advanced tasks such as SLAM.

Recent advancements in deep learning have prompted the integration of DNN to improve the performance of visual SLAM. Cong et al. [14] proposes an algorithm that improves the performance of SLAM in indoor environments by utilizing depth maps from an RGB-D camera and rejecting edge regions of dynamic objects through segmentation results from version 5 of YOLO [15]. However, this approach is better suited for indoor environments with numerous objects such as offices. In this paper, we argue that employing a DNN to replace the depth map of an RGB-D camera is effective for implementing visual SLAM in larger spaces, as demonstrated by the following studies. Jin et al. [16] proposed an algorithm that combines the depth network based on DenseNet [17] with the ORB-SLAM [18] pipeline, including ORB feature extraction and bundle adjustment optimization. Li et al. [19] not only use a ResNet [20]-based depth network but also introduce a similarity-based filter [21] for surfel denoising, accounting for errors that may arise during depth estimation and surfel registration. Existing SLAM algorithms that utilize neural networks often lack a comprehensive consideration of depth estimation techniques and rely on rule-based filtering methods. In this paper, we propose a method that enables adaptive filtering based on the uncertainty of depth estimation, demonstrating experimentally that utilizing the filtered depth map can enhance the performance of VIO.

Kendal et al. [22] addressed foundational concepts for uncertainty research in the field of deep learning. Methods for estimating uncertainty in depth estimation include post-processing techniques for analyzing pre-trained networks and predictive estimation methods that involve designing separate uncertainty estimation networks and incorporating them into the depth network training process. Hornauer et al. [23] defined an auxiliary loss as the mean squared error (MSE) between the estimated depth map of the original input image and the estimated depth map of the side-flipped image after flipping it back. Based on this, an uncertainty map was generated using the gradient of the intermediate layer with respect to the loss. Poggi et al. [24] similarly defined the uncertainty as the absolute error between two outputs generated using the same method and generated an uncertainty map. Additionally, this study applied previously proposed uncertainty estimation methods [25,26,27,28,29,30,31] to depth estimation and analyzed their effectiveness. Eldesokey et al. [32] and Su et al. [33] proposed uncertainty estimation networks to enhance, respectively, depth completion for sparse depth maps and depth estimation from multi-view stereo inputs. In contrast to existing predictive uncertainty estimation methods, we propose a novel approach to defining uncertainty and generating ground truth uncertainty to directly affect the network training process.

## 3. Methodology

This section presents the details of the proposed VIO method. First, we explain the architecture of the DNN in UD-Net for depth estimation and introduce the novel loss function for training the UD-Net. Next, we explain the VIO process that utilizes the estimation results from UD-Net, which was based on VINS-RGBD [9].

### 3.1. Uncertainty-Aware Depth Network

**Figure 2:**
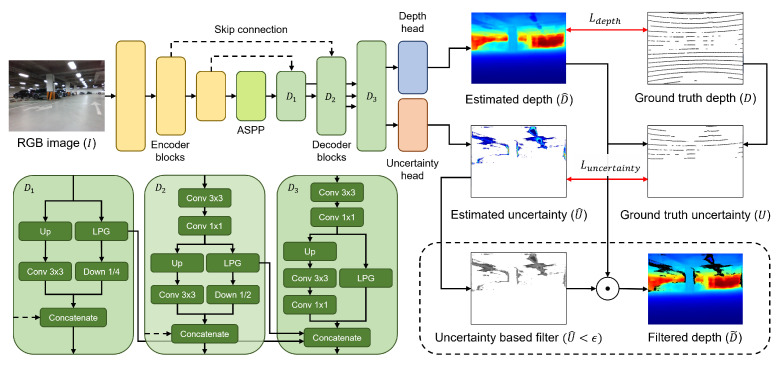
Proposed depth estimation pipeline of UD-Net. Conv 1 × 1 and Conv 3 × 3 refer to convolution blocks that combine 1 × 1 and 3 × 3 convolutional kernel operations, respectively, with the ELU activation function. Up and Down *n* represent 2× upsampling and *n*-times downsampling, respectively. The dotted rounded box indicates the post-processing step that utilizes the output of DNN. ⊙ denotes the element-wise product. Since the valid points in the ground truth depth map are sparse compared to the image resolution, we expanded them using a 5 × 5 kernel for visualization. In the depth map, blue indicates closer distances, while red represents farther distances.

In this paper, among the recently proposed depth network candidates [11,12,13], we selected BTS [11] based on the experiments conducted in Section 4.2. As shown in Figure 2, the DNN for the simultaneous estimation of depth and uncertainty adopts a simple encoder–decoder structure. The encoder leverages DenseNet [17], which is widely known for its efficient feature extraction capabilities. The final output of the encoder passes through an atrous spatial pyramid pooling (ASPP) module [34] to expand the receptive field and is then fed into the first decoder block $D_{1}$. ASPP is composed of five convolution blocks, each containing a 1 × 1 convolution, a 3 × 3 convolution with a distinct dilation rate r∈{3,6,12,18,24}, and ReLU as the activation function. Each decoder block is dual branch, incorporating an LPG layer [11], and it receives inputs not only from the output of the previous decoder block but also from the output of the encoder with the same spatial resolution via a skip connection. The LPG layer is designed based on the geometric assumption that adjacent regions in the image exist on the same plane in 3D space, and its output resolution matches the resolution of the input image. We concatenate the outputs from the LPG layers of $D_{1}$ and $D_{2}$, along with the outputs from the LPG layer of $D_{3}$ and the branch composed of convolutions. The final feature map is fed into the respective heads for depth and uncertainty estimation. The depth head consists of a convolution layer with a 3 × 3 kernel and a sigmoid function, multiplying by a predefined maximum depth value to estimate the depth map. Since the uncertainty of depth is defined in the range of [0,∞] in this paper, the uncertainty head employs a convolution layer with a 3 × 3 kernel and ReLU as the activation function.

Depth estimation becomes increasingly challenging as distance increases, making it prone to errors regardless of the capability of network or the quality of the dataset. The SIlog loss $L_{silog}$ [10] adopted widely in recent supervised learning-based depth estimation methods addresses this issue by being defined on a log scale of relative errors.

(1) $$L_{silog}=\frac{1}{n}\sum_{i}e_{i}^{2}-\frac{\lambda }{n^{2}}{\left(\sum_{i}e_{i}\right)}^{2},$$

where $e_{i}=\log{\hat{d}}_{i}-\log{\hat{d}}_{i}$, ${\hat{d}}_{i}$ and $d_{i}$ represent the *i*-th pixel values in the estimated depth map $\hat{D}$ and the ground truth depth map *D*, respectively. To ensure that the aleatoric uncertainty arising from heteroscedastic noise [22], which varies with the input, is included in the estimated uncertainty, we define the following data-dependent uncertainty-aware depth loss:

(2) $$L_{depth}=\frac{1}{n}\sum_{i}{\left(\frac{e_{i}}{1+{\hat{u}}_{i}}\right)}^{2}-\frac{\lambda }{n^{2}}{\left(\sum_{i}\frac{e_{i}}{1+{\hat{u}}_{i}}\right)}^{2},$$

where ${\hat{u}}_{i}$ represents the *i*-th pixel value in the estimated uncertainty map $\hat{U}$, and the variance focus weight λ is set to 0.85 in our study.

In this study, for error-based uncertainty estimation, we define the ground truth uncertainty $u_{i}$ as the absolute value of the relative error $|e_{i}|$ of depth estimation. The uncertainty loss $L_{uncertainty}$ is defined as a combination of two terms: one that directly compares the ground truth uncertainty with the estimated uncertainty and another that encourages the estimated uncertainty to converge toward zero, which is calculated as follows:

(3) $$L_{uncertainty}=\sqrt{\frac{1}{n}\sum_{i}{\left(u_{i}-{\hat{u}}_{i}\right)}^{2}}+\log\left(1+\frac{1}{n}\sum_{i}{\hat{u}}_{i}\right).$$

By utilizing the loss function based on the ground truth uncertainty derived from the depth estimation results, we can capture the uncertainty arising from model architecture and model parameters at the current training step. In a structure where the encoder and decoder are shared, the second term indirectly guides the network to reduce potential errors in its estimation results. The total loss function $L_{total}$ is defined as the weighted sum of these two loss functions, where the loss weight α is determined experimentally and defined as 100×(currentepoch/maximumepoch), which is calculated as follows:

(4) $$L_{total}=L_{depth}+\alpha L_{uncertainty}$$

### 3.2. Visual Inertial Odometry Based on UD-Net

In this study, we argue that if the estimated uncertainty map is well predicted as intended, the filtered depth will retain only the highly accurate regions, which significantly enhance the performance of VIO. Therefore, the following post-processing step is included as the final stage of the UD-Net pipeline.

(5) $$\tilde{D}=\hat{D}⊙\left(\hat{U}<\epsilon \right)$$

where ϵ represents a positive constant less than 1, which is set to $10^{-6}$ in our experiments, and ⊙ indicates an element-wise multiplication. The VIO leveraging the uncertainty-aware depth map $\tilde{D}$ generated by UD-Net is built upon the VINS-RGBD [9] framework. The VIO system utilizes two types of sensor data: IMU data with an output rate of approximately 200–400 Hz and camera images with a frame rate of about 10–30 Hz. Due to the significant difference in output rates between the two sensors, pre-integration is applied, where a sample of IMU data is sampled between each pair of camera frames. Subsequent to pre-integration, inertial odometry is conducted by estimating translation and rotation via the integration of the sampled IMU data—specifically, accelerometer and gyroscope data. For the RGB images, visual odometry is conducted using the perspective-n-points (PnP) algorithm [35,36,37] based on Shi–Tomasi feature extraction [38] and the Kanade–Lucas–Tomasi sparse optical flow algorithm [39] for feature tracking. In this process, the existing RGB image-based VIO method [40] relies solely on tracked 2D features. Consequently, instead of using PnP, it employs structure from motion (SFM), which means that the scale information depends on inertial odometry. In this study, the depth map $\tilde{D}$ estimated by UD-Net provides estimated scale information, enabling a PnP algorithm being more robust for motion estimation than SFM. The depth values of the matched features across different frames are fixed or filtered after depth validation [40] confirms their similarity. For feature points whose depth values are removed during depth validation or uncertainty-based filtering, depth values are estimated using triangulation [41] and are set as variables that can be optimized. Subsequently, visual–inertial initialization [40] is conducted by complementarily utilizing both odometries. Once the initialization is successful, a sliding window-based local VIO process is iteratively carried out. Through the optimization process with loop closing based on the bag of words approach, accumulated localization and mapping errors are corrected. The map is then constructed using an octree structure [42] being efficient for point cloud management. In the case of the existing method [9], the valid range of depth depends on the RGB-D camera used and is generally constrained to approximately 20 m. However, in the proposed pipeline, it can be applied to broader spaces depending on the performance of depth estimation. Experiments demonstrated that there was a significant performance improvement in broader spaces, such as an indoor parking lot, compared to smaller indoor environments like offices.

## 4. Experimental Results

### 4.1. Experiment Setting and Evaluation Measures

All experiments in this study were conducted using a workstation equipped with an AMD EPYC 7313P 16-Core processor and two NVIDIA GeForce RTX 4090. We utilized the model parameters directly trained for comparison with existing depth estimation methods. For AdaBins, we used the AdamW optimizer [43] with a weight decay of $10^{-2}$ and set the learning rate to $3.5\times 10^{-4}$ [13]. For BTS, NewCRFs, and the DNN of the proposed UD-Net, the Adam optimizer [44] with a weight decay of $10^{-2}$ was employed with a learning rate of $10^{-4}$ [11,12]. Following the baseline network [11], the initial parameters of the encoder and decoder in the proposed DNN were set to DenseNet [17] pre-trained parameters on ImageNet 1K [45] and initialized using the method proposed by Glorot et al. [46]. In the experiments on the underground parking lot dataset, transfer learning was applied to enhance performance and prevent striped noisy patterns [47], which can arise from the sparse ground truth. The model was first trained on the NYUv2 depth dataset [48], and the learned parameters were then used as initial parameters with the learning rate set to $10^{-6}$. To prevent overfitting, random rotations within a range of [−1.0, 1.0] degrees were applied to the KITTI dataset [8], and those within a range of [−2.5, 2.5] degrees were applied to the NYUv2 depth [48] and underground parking lot datasets during training. Additionally, color augmentation, including horizontal flipping and adjustments in contrast, brightness, and color space within the range of [0.9, 1.1], was applied with a 50% probability.

Following previous work on depth estimation [10], we compared the performance of our method against existing supervised methods [11,12,13] using six error metrics and three accuracy metrics. The error metrics, including SIlog, absolute relative error (AbsRel), square relative error (SqRel), root mean squared error (RMSE), root mean square of the inverse depth (RMSEi), and logarithmic error (log10), indicate higher performance with lower values. The accuracy metrics represent the percentage of pixels where the relative error δ, computed as $\max(\hat{d}/d,d/\hat{d})$, is below thresholds of [1.25, $1.25^{2}$, $1.25^{3}$]. Detailed formulas and explanations of the metrics used for evaluating the performance of depth estimation can be found in the work of Eigen et al. [10]. For evaluating the performance of VIO algorithms, we generated ground truth trajectories using the accurate LiDAR-based SLAM algorithm Faster-LIO [49]. To evaluate the performance of VIO, we utilized three error metrics, including the translation and rotation errors of relative pose error (RPE) and the RMSE of absolute trajectory error (ATE) [50]. In the experimental tables, bold values indicate the highest performance, while underlined values represent the second highest performance.

### 4.2. Experimental Results on the KITTI Dataset

The KITTI dataset [8] is widely used not only for depth estimation but also for developing and evaluating computer vision technologies for autonomous driving, such as stereo matching, optical flow, object detection, object tracking, and semantic segmentation. We employed the standard experimental setup for depth estimation as proposed by Eigen et al. [10]. The Eigen split consists of 39,810 images for training, 4424 images for validation, and 697 images for evaluation. To train on images of slightly different sizes, we applied random cropping to a resolution of 704×352. The ground truth depth maps in the KITTI dataset include both the original KITTI depth maps [8], which are LiDAR-based, and the improved KITTI depth maps [51], which are generated through DNN-based interpolation. In this study, we utilized the improved KITTI depth maps to train both existing methods and our proposed DNN, and we evaluated the performance using both types of ground truth.

**Figure 3:**
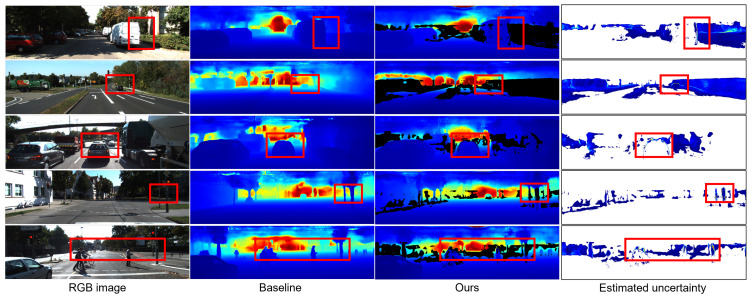
Qualitative results of depth estimation on the KITTI dataset. Each column sequentially presents the input image, the depth map estimated by the baseline depth network, and the depth map and uncertainty map estimated by our method. In the depth and uncertainty maps, blue indicates lower values, while red indicates higher values. Regions with the highest estimated uncertainty for each image are highlighted with a red box. In the depth map, blue indicates closer distances, while red represents farther distances.

Table 1 presents the quantitative results of depth estimation on the Eigen split [10]. We adopted BTS [11], which demonstrated the highest performance across all metrics on both the original and improved KITTI datasets, as the baseline of DNN. Before applying uncertainty-based filtering in UD-Net, the depth estimation results demonstrated comparable or superior performance to the baseline across five error metrics and three accuracy metrics. Since the model demonstrated performance improvements across most metrics without the addition of elaborately designed modules, it indicates that the use of the proposed loss function for training was effective and meaningful. Although there was no improvement in terms of AbsRel, the difference was minimal as 0.001, and our proposed algorithm offers the additional advantage of uncertainty estimation. Applying filtering based on the estimated uncertainty results in a reduction of the regions considered for evaluation; however, this approach effectively addresses areas with significant errors. Specifically, the error was reduced 50.4 percent for SqRel, and the accuracy metric with a threshold of 1.25 showed an increase in the percentage of valid pixels by 4.0 and 2.2 percentage point for the original and improved KITTI, respectively. Figure 3 presents the qualitative results of depth estimation, including both the depth estimation results and the uncertainty estimation results. High uncertainty was observed in objects such as foreground vehicles, dense vegetation, people, and streetlights with colors similar to the background. These areas are prone to noise in LiDAR-based ground truth data or are susceptible to errors in depth estimation using DNNs.

### 4.3. Experimental Results on the Underground Parking Lot Dataset

**Figure 4:**
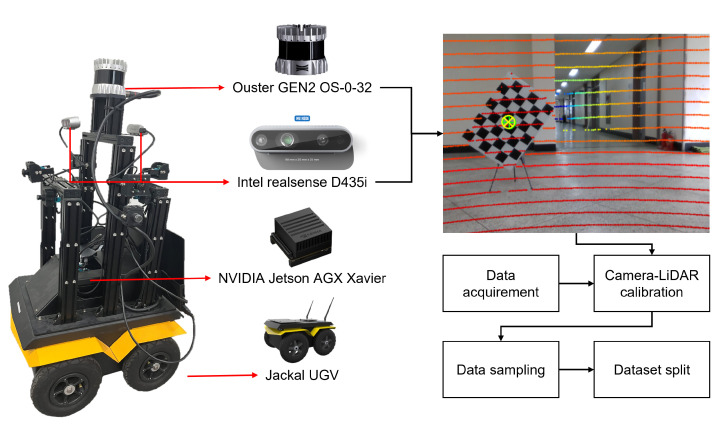
Sensor configuration of mobile robot and dataset construction process for depth estimation dataset. In the projection of LiDAR points, red indicates closer distances, while blue represents farther distances.

A parking lot is an accident-prone environment where both vehicle driving and pedestrian walking occur simultaneously, requiring careful consideration for autonomous driving implementations. Indoor parking lots, in particular, are advantageous for VIO due to their abundance of feature points and finite distances compared to outdoor environments. To regard the application of the proposed algorithm in autonomous parking, we collected a dataset in an underground parking lot using the mobile robot depicted in Figure 4. The mobile robot, based on the Jackal unmanned ground vehicle, was equipped with an Ouster LiDAR and an Intel RGB-D camera sensor, and it utilized an NVIDIA embedded board for sensor data processing and mobility control. The dataset for depth estimation and VIO was acquired under different dates and driving scenarios. To generate the ground truth depth map, we performed calibration to estimate the extrinsic parameters between the camera and LiDAR through off-line calibration [52]. We projected the LiDAR points onto the image plane using the extrinsic parameters. The dataset, consisting of RGB images with a resolution of 640×480 and corresponding ground truth depth maps, was split into 14,424 pairs for training and 5846 pairs for testing.

**Figure 5:**
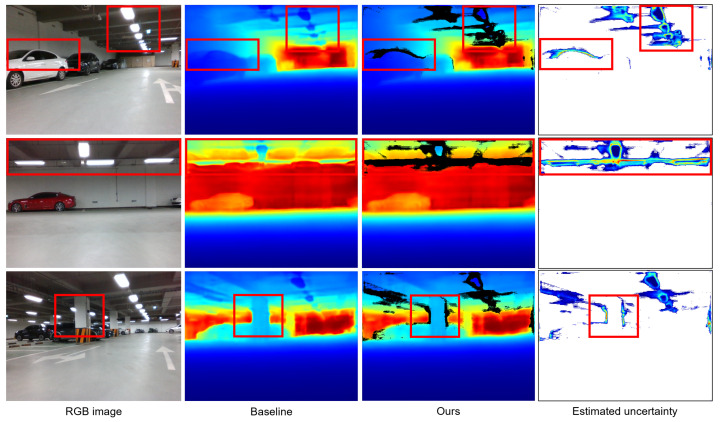
Qualitative results of depth estimation on the underground parking lot dataset. Each column sequentially presents the input image, the depth map estimated by the baseline depth network [11], and the depth map and uncertainty map estimated by our method. In the depth and uncertainty maps, blue indicates lower values, while red indicates higher values. Regions with the highest estimated uncertainty for each image are highlighted with a red box. In the depth map, blue indicates closer distances, while red represents farther distances.

As shown in Table 2, the ranking of depth estimation performance for existing methods [11,12,13] on the underground parking lot dataset differs from that on the KITTI dataset [8]. While BTS [11], which performed best on all metrics in the KITTI dataset, exhibited the lowest performance in the underground parking lot dataset, AdaBins [13] achieved the highest performance with a substantial margin. The depth estimation results from UD-Net prior to uncertainty-based filtering showed improved performance over the baseline [11], but they were still lower compared to other existing methods such as AdaBins [13] and NewCRFs [12]. However, after applying filtering, ours demonstrated the highest performance on six metrics and the second highest performance on three metrics. Figure 5 represents that high uncertainty is estimated in areas prone to depth estimation errors, such as complex structures including pipes and lighting fixtures on the ceiling and pillar. In feature-based VIO, such object boundaries are often targeted for feature extraction. The proposed VIO pipeline employs triangulation and depth validation instead of relying on potentially inaccurate depth estimation results, enhancing robustness.

**Figure 6:**
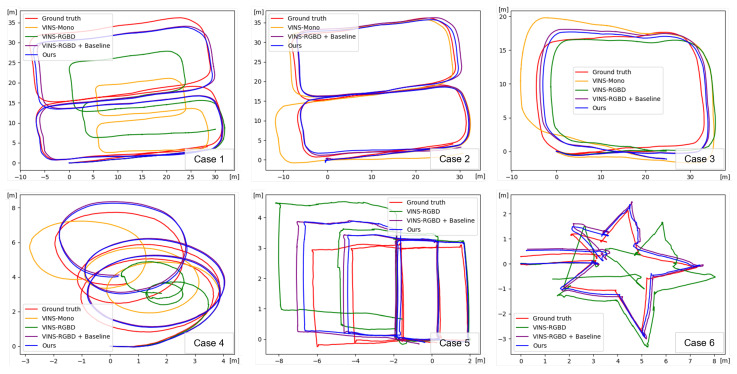
Qualitative results of odometry estimation on the underground parking lot dataset. In case 2, VINS-RGBD, and in cases 5 and 6, VINS-Mono are excluded from the qualitative performance comparison due to significant errors in their estimates.

The dataset for VIO was collected across six scenarios in two categories. Table 3 and Figure 6 present the quantitative and qualitative results for VIO across cases 1 through case 3, which cover three general driving scenarios. Cases 4 through 6 are three specialized driving scenarios: continuous rotation, combined individual rotation and translation, and repeated rotations with acute and obtuse angles. As shown in Table 3, VINS-RGBD [9] exhibits higher performance in the ATE than VINS-Mono [40] for the underground parking lot dataset, except in case 2. In case 2, which involves the longest driving distance, significant errors resulted in a higher average of error metrics. This highlights the need for performance improvements, as such errors could lead to accidents in practical autonomous driving applications. Despite being an RGB-based VIO pipeline, our proposed method consistently outperforms VINS-RGBD [9] across all metrics in every case. When using our proposed VIO pipeline, performance improved in terms of ATE compared to baseline depth estimation [11]. Ultimately, we argue that combining depth estimation with VIO is effective in environments that exceed the ideal maximum depth of RGB-D cameras, and that enhancing depth estimation performance directly contributes to the improved performance of VIO.

## 5. Conclusions

This paper proposes a method to enhance the performance of VIO by integrating DNN-based depth estimation with data from camera and IMU sensors. The proposed UD-Net simultaneously estimates depth maps from RGB images and predicts regions prone to errors in the estimated depth maps. The approach includes a complementary loss function for depth and uncertainty during training and applies uncertainty-based filtering. We observed enhanced depth estimation performance on the public KITTI dataset and evaluated VIO performance using data collected from an underground parking lot environment. Our findings suggest that in environments where the ideal maximum depth range provided by RGB-D cameras is exceeded, well-trained DNN-based depth estimation can significantly enhance VIO performance. Consequently, depth estimation serves as an effective alternative or complement to distance-constrained RGB-D cameras and expensive LiDAR systems, depending on the application environment.

Our proposed method significantly improves the accuracy of depth estimation and odometry estimation. However, the computational resources required, along with the resolution of the depth neural network and input images, may pose challenges for real-time application. Therefore, future work should focus on optimizing the deep neural network to enhance real-time applicability. Furthermore, the generalization performance, which is often overlooked in the current field of supervised depth estimation, is an essential aspect that must be considered when applying these methods in practical applications. To address this issue, we are considering the utilization of foundation models or unsupervised depth estimation approaches in future work. However, this study demonstrates that expensive LiDAR sensors can be replaced with depth networks in the implementation of SLAM, and that enhancing the reliability of the depth network directly contributes to improvements in odometry estimation.

## Figures and Tables

**Table 1:** Quantitative results of depth estimation on the KITTI dataset. Error metrics highlighted in red indicate that lower values are better, while accuracy metrics highlighted in blue indicate that higher values are better. † indicates the results before applying the filtering process.

Method	Error Metric ↓						Accuracy Metric ↑		
	**AbsRel**	**SqRel**	**RMSE**	**RMSEi**	**SIlog**	**log10**	δ<1.25	$\delta <1.25^{2}$	$\delta <1.25^{3}$
Original KITTI [8]									
BTS [11]	0.084	0.563	4.096	0.176	16.624	0.040	0.905	0.965	0.983
NewCRFs [12]	0.117	0.786	4.750	0.208	19.596	0.054	0.845	0.946	0.977
AdaBins [13]	0.102	0.636	4.102	0.186	17.718	0.046	0.879	0.958	0.982
UD-Net ^†^	0.085	0.547	4.037	0.173	16.425	0.040	0.905	0.967	0.983
UD-Net (ours)	**0.061**	**0.276**	**2.674**	**0.127**	**11.980**	**0.028**	**0.945**	**0.982**	**0.991**
Improved KITTI [51]									
BTS [11]	0.060	0.255	2.821	0.097	8.967	0.027	0.954	0.992	0.998
NewCRFs [12]	0.090	0.462	3.744	0.140	12.782	0.040	0.901	0.979	0.995
AdaBins [13]	0.074	0.336	2.939	0.112	10.337	0.031	0.937	0.988	0.997
UD-Net ^†^	0.061	0.250	2.784	0.097	8.960	0.027	0.954	0.993	0.998
UD-Net (ours)	**0.046**	**0.126**	**1.816**	**0.072**	**6.506**	**0.020**	**0.976**	**0.997**	**0.999**

**Table 2:** Quantitative results of depth estimation on the underground parking lot dataset. Error metrics highlighted in red indicate that lower values are better, while accuracy metrics highlighted in blue indicate that higher values are better. † indicates the results before applying the filtering process.

Method	Error Metric ↓						Accuracy Metric ↑		
	**AbsRel**	**SqRel**	**RMSE**	**RMSEi**	**SIlog**	**log10**	δ<1.25	$\delta <1.25^{2}$	$\delta <1.25^{3}$
NewCRFs [12]	0.094	0.377	1.797	0.143	13.914	0.035	0.913	0.977	**0.993**
AdaBins [13]	**0.082**	**0.338**	1.827	0.137	13.243	**0.032**	0.928	0.978	0.992
BTS [11]	0.102	0.444	1.972	0.155	14.497	0.038	0.908	0.973	0.991
UD-Net ^†^	0.101	0.436	1.944	0.153	14.273	0.038	0.910	0.973	0.991
UD-Net (ours)	0.086	0.359	**1.765**	**0.135**	**12.485**	**0.032**	**0.937**	**0.981**	0.992

**Table 3:** Quantitative results of odometry estimation on the underground parking lot dataset. Baseline mean BTS [11] which is the basis of depth network. † indicates the results before applying the filtering process.

Driving Scenario		Case 1	Case 2	Case 3	Case 4	Case 5	Case 6	Average
**Driving Distance [m]**		**225.35**	**225.35**	**122.26**	**44.32**	**44.27**	**26.02**	
Method	Depth	RMSE of ATE [m]						
VINS-Mono [40]	None	7.6614	2.0864	2.7397	0.8252	2.9859	4.6489	4.1034
VINS-RGBD [9]	Sensor	5.8164	7.8056	0.9733	1.5514	0.7652	0.7833	4.8166
Ours	Baseline	2.0736	0.8750	0.6901	0.2468	0.3556	0.2698	1.1382
	UD-Net ^†^	**2.0724**	0.8809	0.7417	**0.2121**	0.2948	0.2277	1.1411
	UD-Net (ours)	2.1260	**0.7693**	**0.5627**	0.2193	**0.2507**	**0.2037**	**1.0870**
Method	Depth	Translation error of RPE [m]						
VINS-Mono [40]	None	0.1518	0.0539	0.0563	0.0857	0.3136	0.2527	0.1127
VINS-RGBD [9]	Sensor	0.1217	0.5044	0.0753	0.2152	0.0632	0.1252	0.2413
Ours	Baseline	**0.0367**	**0.0328**	0.0302	**0.0304**	0.0368	0.0562	**0.0346**
	UD-Net ^†^	0.0368	0.0331	0.0305	0.0311	0.0365	0.0565	0.0348
	UD-Net (ours)	0.0377	0.0330	**0.0295**	0.0308	**0.0338**	**0.0547**	0.0347
Method	Depth	Rotation error of RPE [deg]						
VINS-Mono [40]	None	**0.1379**	**0.1442**	0.1844	0.4872	0.5463	**0.6791**	**0.2175**
VINS-RGBD [9]	Sensor	0.3601	0.4537	0.3722	1.3643	1.2669	2.4969	0.5969
Ours	Baseline	0.1396	0.1576	**0.1828**	0.4958	0.5164	0.7093	0.2220
	UD-Net ^†^	0.1405	0.1649	0.1894	0.4926	0.5169	0.7133	0.2258
	UD-Net (ours)	0.1394	0.1557	0.1870	**0.4863**	**0.5102**	0.7233	0.2216

## Data Availability

The KITTI dataset is publicly available online. The public dataset can be found at https://www.cvlibs.net/datasets/kitti, accessed on 29 August 2024.
